# Healthcare Professionals’ Perspectives on the Implementation of the Integrated Care Models for Chronic Patients

**DOI:** 10.7759/cureus.42173

**Published:** 2023-07-20

**Authors:** Nikolaos V Konstantinidis, Michael A Talias

**Affiliations:** 1 Healthcare Management/Health Economics, Open University of Cyprus, Nicosia, CYP

**Keywords:** healthcare professionals, care coordination, applicability index, healthcare services, chronic care model, policy reforms, survey study, greece, chronic diseases, integrated care

## Abstract

Objective

This study aimed to assess the understanding, adoption, and barriers to implementing integrated care for chronic diseases among healthcare professionals in Greece. By gathering insights from healthcare professionals directly involved in the care of patients with chronic conditions, this study sought to identify areas for improvement and inform future policy and strategic initiatives to enhance the quality of care and patient outcomes in Greece. Specific objectives included assessing healthcare professionals' knowledge and understanding of integrated care concepts, principles, and components in chronic disease management and exploring healthcare professionals’ experiences in providing or participating in integrated care activities for patients with chronic diseases.

Methods

This study employed a census-based survey design to assess healthcare professionals' understanding, adoption, and barriers to the implementation of integrated care for chronic diseases in the Greek healthcare system. The sampling technique has been used to ensure the representation of different healthcare professions and regions in Greece. The survey questionnaire was structured based on the internationally recognized Chronic Care Model Elements Survey, specifically tailored to capture insights on integrated care for chronic illnesses in Greece. Healthcare professionals from diverse settings, including primary healthcare centers, public and private hospitals, specialty clinics, rehabilitation centers, home-based care services, and private sector practitioners, were targeted to gather comprehensive perspectives. Both urban and rural areas were included to ensure a representative sample, enabling an understanding of integrated care implementation in Greece.

Results

A total of 246 responses from healthcare professionals in Greece were collected and analyzed. An applicability index was constructed to evaluate the suitability of the integrated care system in Greece, utilizing variables collected during the survey. The reliability of the index was assessed using Cronbach's Alpha coefficient, which demonstrated a high value of 0.940, indicating strong internal consistency and correlation among the questions related to integrated care. However, the data collected for Greece exhibited a departure from a normal distribution using the Shapiro-Wilk test, suggesting the presence of barriers to the implementation of integrated care within the Greek healthcare system.

Conclusions

The study revealed several obstacles to integrated care implementation, encompassing organizational and individual factors, such as financial constraints, cultural differences, and regulatory challenges. Tackling these barriers will require a collective approach and close collaboration among multiple stakeholders to create an enabling context for adopting integrated care. Possible strategies involve resource allocation, fostering communication and cooperation among healthcare providers, and revising regulatory frameworks to facilitate integrated care practices. In order to achieve the national objectives of improving public health, the survey increases the focus on evidence-based public health.

## Introduction

Chronic diseases are among the leading causes of morbidity and mortality worldwide, with a significant impact on the quality of life of individuals and the overall burden on healthcare systems. According to the World Health Organization (WHO), chronic diseases account for over 70% of all deaths globally, significantly impacting healthcare systems, quality of life, and economic development. In Greece, the healthcare system faces numerous challenges in managing chronic diseases, including fragmentation, limited resources, and a need for coordination among various healthcare providers. Despite ongoing efforts to restructure and modernize the Greek healthcare system, the implementation of integrated care remains limited, and its potential to improve the management of chronic diseases has yet to be fully realized [1-3].

In Greece, the management of chronic diseases has primarily been fragmented. This fragmentation often leads to suboptimal patient outcomes, increased healthcare costs, and inefficient resource use. To address these challenges, there is a growing recognition of the need for a more integrated approach to managing chronic diseases [4].

However, the implementation of integrated care models is challenging. It requires changes at multiple healthcare system levels, from policy and organizational changes to practice patterns among healthcare professionals. Understanding the attitudes and perceptions of healthcare professionals towards integrated care is crucial, as their acceptance and willingness to change their practice are critical determinants of successful implementation [5-7].

This study aims to assess the attitudes and perceptions of healthcare professionals in Greece towards implementing an integrated care perspective for chronic diseases. It will identify the perceived barriers and facilitators for implementing the integrated care model and evaluate the willingness and readiness of healthcare professionals to adopt this new model of care. The findings of this study will provide valuable insights to inform strategies for the successful implementation of integrated care for chronic diseases in Greece.

Given these considerations, this study holds significant implications for policymakers, healthcare managers, and practitioners. It can inform decision-making and practice in the quest for more effective and efficient management of chronic diseases, ultimately contributing to improved patient outcomes and health system sustainability.

Finally, this study can contribute to the broader discourse of implementing integrated care for chronic diseases. The lessons learned from the Greek context can provide valuable insights for other countries on a similar journey towards integrated care.

## Materials and methods

This study used a census-based survey design to investigate the attitudes and perceptions of healthcare professionals toward integrated care for chronic diseases in Greece. A census analysis refers to the process of examining and interpreting data collected from a census. A census is a systematic collection of demographic, social, and economic information among healthcare professionals. The goal of census analysis is to provide insights into the characteristics, composition, and trends within healthcare professionals.

Study location and setting

This study was conducted in various healthcare settings across Greece, including urban and rural areas, to capture diverse perspectives on integrated care for chronic diseases. The healthcare settings targeted in the survey included primary healthcare centers, private and public hospitals, specialty clinics focused on managing specific chronic conditions, rehabilitation centers, home-based care services, and professionals from the private sector. The survey was conducted between May 2021 and May 2022. The survey was distributed only by electronic means to professionals in health. The selection was made from databases of medical associations, professional associations, and databases for commercial purposes. The distribution of the questionnaire to private hospitals, public hospitals, and health centers was delivered by the agencies themselves because it is forbidden to give the researcher the emails of health professionals. The questionnaire was distributed to the sampled healthcare professionals via various online channels, including email and professional networking platforms. Participants were given a transparent description of the study's objectives, and informed consent was obtained accordingly.

Inclusion criteria

To be eligible for participation in the survey study, criteria relevant to the study have been established. These include participants who must be healthcare professionals, currently practicing in a healthcare setting in Greece, be at least 18 years old, and provide informed consent to participate in the study. We have carefully selected these inclusion criteria to ensure the relevance, reliability, and applicability of the study's findings within the Greek context. By doing so, our research aims to provide valuable insights that contribute to the ongoing endeavors in enhancing integrated care for chronic disease management.

Exclusion criteria

Exclusion criteria that disqualify prospective participants from inclusion, including retired participants, have been adopted. Therefore, healthcare professionals who did not practice, who were retired or non-healthcare professionals, and those practicing outside of Greece or those who did not provide informed consent have been excluded from the study.

Instrument

The primary instrument for this study has been an online survey questionnaire, which was developed to assess healthcare professionals' understanding, adoption, and barriers to implementing integrated care for chronic diseases in Greece. A sampling technique has been used to ensure the representation of different healthcare professions and regions in Greece. The structure of the questionnaire was based on an internationally recognized questionnaire on chronic diseases, the Chronic Care Model Elements Survey [8,9]. It refers to an assessment tool that focuses on the elements of the Chronic Care Model to evaluate the implementation and effectiveness of chronic care management practices in healthcare settings. The CCM is a widely recognized and evidence-based framework for improving the care of patients with chronic illnesses, emphasizing the importance of a well-organized, proactive approach. The survey included questions related to the six critical components of the regular care model. The survey was hosted on a secure online platform, Google Forms. The questions have been designed to be clear, concise, and non-leading. By drawing upon the insights provided by experts and examining the existing body of literature, the researchers developed a questionnaire that encompassed the key aspects of integrated care for chronic diseases. All responses were collected anonymously to ensure confidentiality.

Data analyses

The data collected from the questionnaires were analyzed using descriptive and inferential statistical methods to draw meaningful insights about healthcare professionals' understanding, adoption, and barriers to implementing integrated care for chronic diseases in Greece. The analysis was performed using the statistical software SPSS (IBM Corp., Armonk, NY). The data analysis process comprised data cleaning and preparation to ensure accuracy and consistency, descriptive statistics, bivariate analysis, multivariate analysis, and subgroup analysis to explore the data more deeply, focusing on specific subgroups of interest. Finally, the results of the data analysis were interpreted in the context of the study's objectives and the existing literature on integrated care for chronic diseases.

Ethical considerations

The study has been subject to several ethical considerations (IRB approval is EEBK EP 2021.01.235 from Cyprus National Bioethics Committee) to ensure that the rights of the participants are respected. Before starting the survey, participants were informed about the purpose of the study, the nature of their involvement, that their participation is voluntary, and that they have the right to withdraw at any time without penalty. Participants have been assured that their responses will be anonymous, and that no personally identifiable information will be collected that could link them to their answers.

## Results

Demographics and professional background

The exact number of questionnaires distributed electronically cannot be determined. The reason is that they were distributed exclusively by healthcare public and private providers, most of whom still need to provide such information. In addition, the questionnaires were distributed to private healthcare professionals 1460 in total. Therefore, the data has been evaluated based on the questionnaire responses. The sampling technique used was initially census-based, aiming to study all possible health institutions in Greece. However, in public and private hospitals, sampling permission was given by the hospitals themselves, while in some smaller health centers, permission had to be given by the administrative region. This procedural hurdle resulted in some small health centers not being able to be represented in the research, as they either did not answer the questionnaire or were never given permission to distribute the questionnaire to health professionals.

The sample size included 32 public hospitals, four private hospitals, and 16 health centers. The reasons to exclude small hospitals from a survey for health professionals in Greece in the following.

Sample Representativeness

Small hospitals may have a limited number of healthcare professionals, and their inclusion in the survey may not provide a representative sample of the broader healthcare system in Greece. Excluding small hospitals can help ensure a more comprehensive representation of healthcare professionals across different settings and regions.

Resource Constraints

Conducting a survey in numerous small hospitals can be resource-intensive and time-consuming. By excluding small hospitals, researchers can focus their efforts on larger healthcare facilities that have a higher concentration of healthcare professionals and patient populations, optimizing the use of resources.

Generalizability

The findings of a survey conducted in small hospitals may have limited generalizability to the larger healthcare system in Greece. Excluding small hospitals allows researchers to gather insights from healthcare professionals working in larger, more diverse settings, which may better reflect the overall challenges and experiences within the healthcare system.

Statistical Power

Excluding small hospitals may help ensure an adequate sample size in each category, allowing for more statistically robust analyses and meaningful interpretation of the survey results.

Policy Impact

When designing surveys for policy evaluation or reform purposes, focusing on larger hospitals can provide insights that are more likely to have a substantial impact on policy decisions due to their scale and influence within the healthcare system.

A total of 246 responses have been collected, and no questions remain unanswered. The demographic characteristics of healthcare professionals are 52% women and 48% men, while 39% live in Athens, 23.6% in Thessaloniki, and 37.4% in other cities in Greece. Participants are 41.5%, those between 46 and 55, 26% between 56 and 65, 21.5% between 36 and 45, 8.1% between 25 and 36, and 2.8% those 66 to 67. Based on their professional occupation, 62.6% are public servants in public healthcare providers, 11.4% are working in private healthcare providers, and 26% are self-employed. In addition, 60.6% are doctors, 31.3% are nurses, 2.4% are psychologists, 2.4% are social workers, and 1.2% are physiotherapists, while 2% reported “other.” Among the doctors, 2.9% are cardiologists, 22.1% are pathologists, 9.1% are surgeons, 6.5% are psychiatrists, 6.5% are otolaryngologists, 5.2% are neurologists, 3.2% are gastroenterologists, and 4.5% reported “other.” Regarding education, 38.2 have a master’s, 24.8% have a PhD, and 4.5% have a postdoc. Also, 67.9% have special knowledge (refers to a specific and in-depth understanding of how to effectively handle or treat long-term health conditions. about the management of chronic diseases) while 32.1% reported a lack of knowledge. In addition, 79.3% said they attended seminars, conferences, or workshops, and 20.7% reported being negative.

Main outcome

In the present analysis, health professionals in Greece were studied regarding implementing the integrated care system and the parameters that affect this application. The dimensions quantified concern the management of chronic diseases by leadership and management, the management of patients with chronic conditions, the care of patients with chronic diseases, some information on patients with chronic diseases, and relevant education and views on the management system for patients with chronic illnesses.

In addition, a set of variables from Greece was collected, creating an applicability index of the integrated care system. More specifically, 17 questions were used from the shared questionnaire, which gave Cronbach's Alpha 0.940 [10,11]. This score was then checked for regularity through the Shapiro-Wilk test [12,13], which showed that the data for Greece do not follow a normal distribution (p<0.001).

The tables show the distribution of responses to each question related to the six critical components of the chronic care model. Table 1 studies the views of participants working in Greece regarding managing chronic diseases through the leadership and management of health structures.

**Table 1:** Frequency distribution and percentages of responses to questions about the health system organization – component of the chronic care model.

		N=182 (246 minus 64 self-employed)	%
Q1	Is there evidence of leadership in your organisation regarding managing chronic diseases?		
	There are none, and there needs to be more interest.	99	54.4
	They exist in the strategic or action plans but need more resources.	27	14.8
	It is written down in a strategic or action plan, and the resources are available.	33	18.1
	They are part of a long-term strategy and have been appointed certain public servants responsible for its implementation.	23	12.6
Q2	The objectives of the organisation for chronic diseases		
	…do not exist or exist to a minimal extent.	115	63.2
	... they exist but have not been refinanced.	16	8.8
	... they are measured and renewed every year.	37	20.3
	... they are measured and renewed yearly, and there is a keen interest in increasing them.	14	7.7
Q3	Service improvement strategies for chronic diseases		
	…do not exist or exist to a minimal extent.	82	45.1
	... exist only for specific diseases.	88	48.4
	... exist and are part of an existing strategic plan.	9	4.9
	... are configured, which leads to an improvement in the services provided.	3	1.6
Q4	The Governor or Director of the organisation		
	…discourages dealing with patients with chronic conditions.	5	2.7
	….does not prioritise dealing with patients with chronic diseases.	67	36.8
	…encourages the improvement of services to patients with chronic diseases.	87	47.8
	…has as a priority dealing with patients with chronic diseases	23	12.6

More specifically, it emerged that most participants, reaching 54.4%, believe there needs to be more evidence of leadership in their organization regarding managing chronic diseases. In addition, 63.2% of respondents report no targets for the organization of chronic illnesses, with 48.4% reporting that there are specific strategies to improve services only for particular diseases and 45.1% reporting that there is no or little interest in such systems. At the same time, 47.8% of respondents seem to believe that the administrator-director encourages the improvement of services for patients with chronic diseases. However, 36.8% of respondents indicate that the administrator-director does not prioritize individuals with chronic conditions [14,15]. A post hoc Bonferroni analysis was conducted in order to reveal statistically significant differences of the applicability score, as for the professional employment status of the participants. As shown (Appendices), the self-employed health professionals, do not significantly differ as for this score with the rest employees (civil or private). So, there is no sign of bias derived from that fact. Table 2 captures health professionals' responses concerning managing patients with chronic diseases.

**Table 2:** Frequency distribution and percentages of responses to questions about delivery system design, community resources, and policies – chronic care model components.

		N=182 (246 minus 64 self-employed)	%
Q5	Is there an interface with health organisations outside your Organization to manage patients with chronic diseases?		
	There is little or no interfacing.	102	56.0
	There are organisations, but the interconnection procedures still need to be completed.	20	11.0
	There are procedures for interfacing with the organisations, but funding is needed.	20	11.0
	There is integrated cooperation between the health system, social organisations and patients.	40	22.0
Q6	Is there an interface with other social service providers to manage patients with chronic conditions?		
	There is little or no interfacing.	102	56.0
	There are agencies, but the interconnection procedures still need to be completed.	44	24.2
	There are procedures for interfacing with the agencies, but funding is needed.	32	17.6
	There is comprehensive cooperation between the health system, agencies and patients.	4	2.2
Q7	Do you know if your organisation has been assessed on the management of chronic diseases in the last five years?		
	No or very little.	126	69.2
	A small report is made, mainly for management reasons.	28	15.4
	The complete report is made at regular intervals.	9	4.9
	It is done at regular intervals.	19	10.4
Q8	Are there health programs at the district level to manage patients with chronic diseases?		
	There is no or little interest.	126	69.2
	There are procedures and programmes which still need to be implemented.	37	20.3
	Some procedures and programs deal with one or two chronic diseases.	17	9.3
	There are procedures and programs for most chronic diseases.	2	1.1

Table 2 studies healthcare professionals' views regarding managing patients with chronic diseases. Most respondents, reaching 56%, state that there is no or, to a small extent, no connection with health institutions outside the organization where they are empty management of patients with chronic diseases. At the same time, 56% of professionals report that there is no or, to a small extent, no connection with other social service providers for managing patients with chronic diseases. In the last five years, 69.2% of the sample states that either no or, to a small extent, the employment agency has been evaluated regarding the management of patients with chronic diseases. Finally, 69.2% of the sample states that there are regional health programs for managing reported patients to a minimal or no extent. Table 3 captures the responses concerning the decision support.

**Table 3:** Frequency distribution and percentages of responses to decision support – components of the chronic care model.

		N=246	%
Q9	Incentives and guidelines for dealing with chronic diseases.		
	They do not exist or exist to a minimal extent.	127	51.6
	They exist and are used to achieve patient purposes.	34	13.8
	They exist and aim to improve services and reduce costs.	44	17.9
	They exist and are used to provide better health services.	41	16.7
Q10	Assessment and guidelines for self-management of needs and activities by patients with chronic diseases.		
	There is no or little interest.	128	52.0
	It is in standard form for most chronic diseases.	17	6.9
	It is under preparation.	74	30.1
	They exist and are in standard form.	27	11.0
Q11	Support for self-management of illness by patients with chronic diseases.		
	It is limited to providing information, leaflets, and advice.	114	46.3
	Reference is made to qualified professionals outside the organisation.	91	37.0
	Qualified clinical professionals of the organisation perform it.	21	8.5
	There is a comprehensive liaison program with experts inside and outside the organisation.	20	8.1
Q12	Are cross-sectoral and interdisciplinary processes for the management of chronic diseases?		
	There is no or little interest.	138	56.1
	Procedures and efforts are being made for available medical specialities.	81	32.9
	Processes and meetings are happening to implement new guidelines and accountability.	22	8.9
	They exist and are part of broader strategic planning.	5	2.0
Q13	Do you know what integrated care is?		
	Not at all or very little.	67	27.2
	We apply some of the principles of integrated care.	142	57.7
	There is an application of integrated care between primary and secondary health care.	12	4.9
	An integrated care system is implemented with social service providers.	25	10.2

Table 3 studies statements related to the care of patients with chronic diseases, according to health professionals working in Greek structures. The most significant number of respondents, 51.6%, report no or minimal incentives and guidelines for dealing with chronic diseases. In addition, 52% of measured believe that there are no, or to a small extent, assessments and procedures for self-management of needs and activities by patients with chronic diseases [14,16]. In addition, 46.3% of those who report that support for the self-management of chronic patients is limited to providing information leaflets and advice, with 37% reporting that it is done with referrals from qualified professionals outside the institution. Moreover, 56.1% reach those who believe that intersectoral and interdisciplinary procedures for managing chronic diseases do not exist or that they live to a minimal extent. In addition, 57.7% of the sample represents those who believe that integrated care for a patient is applied based on some of the principles of integrated care [17-20]. Table 4 captures the responses concerning self-management support.

**Table 4:** Frequency distribution and percentages of responses to self-management support – components of the chronic care model.

		N=246	%
Q14	Do you keep an electronic file for each patient with a chronic disease?		
	No or only minimal data.	83	33.7
	We keep enough data.	89	36.2
	We keep detailed data on his disease.	13	5.3
	We keep full details of his disease and history.	61	24.8
Q15	Do you share the information you have recorded electronically with other organisations or healthcare professionals as part of your duties?		
	No or only minimal data.	185	75.2
	Enough data.	50	20.3
	Detailed facts about his disease.	5	2.0
	Full details about the disease and its history.	6	2.4
Q16	Are electronic protocols and guidelines available for managing patients with chronic disease?		
	No or only minimal data.	140	56.9
	Enough data.	70	28.5
	Detailed data on most chronic diseases.	32	13.0
	Complete data on most chronic diseases.	4	1.6
Q17	Have you been trained to deal with patients with chronic diseases?		
	Not at all or only very little.	82	33.3
	Enough.	116	47.1
	I have comprehensive training in my speciality for chronic diseases.	40	16.3
	In detail for most chronic diseases.	8	3.3

Table 4 explores the information and education the interviewed health professionals in Greece have for patients with chronic diseases. 36.2% of respondents state that enough data is kept in the electronic file for each patient with chronic illness, and 33.7% report that no or little data is saved. However, the vast majority, reaching 75.2%, report that they need to share the information they have recorded electronically with other institutions or health professionals in the context of their duties or share little data. At the same time, 56.9% of health professionals working in Greece state that there are no available electronic protocols and guidelines for managing patients with chronic disease or that there is little corresponding data. Finally, 47.2% of the sample states that they have been trained enough to deal with patients with chronic illnesses. Also, 33.3% are occupied by those trained in this field, very little or no [16,21,22]. Table 5 captures how significantly the degree of applicability index-specific survey parameters are affected.

**Table 5:** Applicable index for integrated care in Greece based on responses from healthcare professionals.

Parameter	Applicability Index of integrated care in Greece
Gender	0.000
Place of residence	0.033
Age	0.089
Professional employment (public healthcare professional, private healthcare professional, self-employed)	0.025
Type of place of employment (public healthcare organisation, private healthcare organisation, private healthcare professional)	0.007
Professional status (Doctor, nurse. Social worker, psychiatrist etc.)	0.004
Doctor’s specialities	0.711
Higher education level (master, PhD, postdoc)	0.004
You acquired special knowledge on managing patients with chronic diseases during your studies.	0.177
Have you attended seminars or conferences, or workshops on chronic diseases?	0.006

## Discussion

The findings of this study provide valuable insights into healthcare professionals' understanding, adoption, and barriers to implementing integrated care for chronic diseases in Greece. This discussion section highlights key findings and their implications for policy, practice, and future research [22-25].

This survey outlines the procedure utilized to develop and assess an applicability index specifically designed for the integrated care system in Greece. Integrated care systems aim to unite various aspects of healthcare, including primary, secondary, and social care, with the objective of offering a comprehensive approach to patient well-being. To create this applicability index, a range of variables pertaining to Greece were gathered. To ensure the dependability of these questions as an accurate measure of the applicability index, the researchers calculated Cronbach's Alpha. A high Cronbach's Alpha value (in this instance, 0.940) signifies a strong correlation and internal consistency among the questions, indicating that they collectively form a reliable measure of the applicability index for the integrated care system in Greece [26-28].

However, a normality test was conducted using the Shapiro-Wilk test to evaluate the data further. The test showed that the data for Greece do not follow a normal distribution (p<0.001), which means the data are not distributed normally and could have potential implications for subsequent statistical analysis. The low p-value (p<0.001) represents that the data's deviation from a normal distribution is statistically significant.

The study identifies several factors that significantly affect the applicability of the integrated care system in Greece, which has important implications for healthcare policymakers and stakeholders. By understanding the role of these factors (gender, place of residence, professional employment, type of employment, professional status, higher education level, and attendance at relevant seminars), targeted strategies and interventions can be developed to improve the implementation and adoption of integrated care systems.

The findings suggest that there may be more than a one-size-fits-all approach to integrated care, and it is essential to consider the diverse needs and characteristics of different population groups when designing and implementing integrated care systems. Policymakers and stakeholders can use this information to inform more inclusive and accessible strategies, such as expanding integrated care services to underserved areas, promoting interdisciplinary collaboration, and utilizing technology to enhance care coordination [23,24].

Based on the data presented, it appears that there may be room for improvement in terms of prioritizing and addressing chronic disease management within the organization (Q1), indicating a potential need for organizations to reevaluate their approach to chronic disease management and develop more comprehensive and actionable objectives to improve the health outcomes for individuals affected by chronic diseases (Q2). Also, implement more inclusive and effective service improvement strategies for chronic diseases, ensuring that all individuals with chronic conditions receive adequate care and support (Q3). Furthermore, it highlights the need for better collaboration and coordination between organizations to provide more comprehensive and effective care for patients with chronic conditions (Q5).

In addition, the data highlight the need for better collaboration and coordination between organizations and social service providers to provide more comprehensive and effective care for patients with chronic conditions (Q6), the need for organizations to implement more robust and systematic evaluations of their established disease management practices to identify areas for improvement and ensure adequate care for patients with chronic conditions (Q7). Also, the importance of increased attention and the implementation of district-level health programs to better address the management of patients with chronic diseases, ensuring more effective and widespread care (Q8). Furthermore, the need for increased focus on providing assessment and guidelines to empower patients to better self-manage their chronic conditions, potentially improving health outcomes and overall quality of life (Q10, Q11); the need for increased collaboration and coordination among different sectors and disciplines (Q12) and the majority of respondents (57.7%) reporting applying some principles of integrated care in their organization (Q13).

To a further extent, there is a need for organizations to improve their information management practices to support better the care and management of patients with chronic conditions (Q14), the importance of improved communication and collaboration between different entities involved in the care and management of patients with chronic diseases (16). Enhanced training can lead to better patient care, improved health outcomes, and a higher quality of life for patients with chronic conditions. Healthcare organizations must invest in ongoing education and training for their staff to effectively manage chronic diseases (Q17).

The Chronic Care Model is an evidence-based framework for improving the management of chronic diseases within healthcare organizations. It emphasizes a proactive, patient-centered approach that incorporates six essential elements: self-management support, delivery system design, decision support, clinical information systems, the organization of healthcare, and community resources. By evaluating the data presented in the context of the CCM, we can identify areas for improvement and potential strategies to enhance chronic disease management [25,26].

Self-Management Support

The data suggest that there is room for improvement in providing support for the self-management of illness by patients with chronic diseases. Healthcare organizations should consider implementing programs and resources to help patients better understand their conditions, develop skills to manage their health and build confidence in their ability to self-manage their chronic diseases [27].

Delivery System Design

Organizations should prioritize establishing cross-sectoral and interdisciplinary processes for managing chronic diseases. By encouraging better communication and collaboration between healthcare professionals, healthcare systems can deliver more coordinated and effective care for patients with chronic conditions.

Decision Support

Healthcare organizations should focus on enhancing the training of healthcare professionals in managing patients with chronic diseases. This includes offering continuing education opportunities, sharing evidence-based guidelines, and promoting a learning and professional development culture.

Clinical Information Systems

The data indicate that many organizations must share electronic patient information with others or healthcare professionals. Developing and utilizing clinical information systems that facilitate the sharing of patient data can help improve care coordination and the overall management of chronic diseases.

Organization of Healthcare

The data suggest that there is a need for healthcare organizations to reevaluate their strategic planning and commitment to addressing chronic disease management. Leaders should prioritize developing and implementing policies and strategies that emphasize comprehensive and proactive care for individuals with chronic conditions.

Community Resources

The data highlight a potential need for more integration between healthcare organizations and community resources. Healthcare organizations should work closely with community partners to ensure that patients with chronic diseases have access to the necessary resources and support services to manage their conditions effectively.

By addressing these areas of the Chronic Care Model, healthcare organizations can develop a more proactive and patient-centred approach to chronic disease management, improving health outcomes and overall well-being for patients with chronic conditions.

In addition, the questionnaire was drawn up to address specific categories of healthcare professionals. The selection of this category was made in order to investigate the supply side in real terms with the greatest possible representativeness. This approach did not concern the demand side as this issue exceeded the limits of this study. The focus on the supply side was done in terms of integrated care, which separates the supply at a micro, medium and long level, but also in horizontal and vertical integration.

Also, the study created profiles for each participant’s views on the place of residence, age, type of work structure, leadership, self-management of needs and activities by patients with chronic diseases and the healthcare system, and specific knowledge for chronic diseases (see Appendix). The contribution of Public Health to human development has as a key parameter the interconnection of scientific knowledge with health programs and policies which will likely be successful in promoting the health of the population. To use science in practice, we need to combine information about evidence-based interventions from the evaluated literature with the dynamics of a particular real environment. Evidence-based public health is crucial because it ensures that public health policies and interventions are based on sound scientific evidence and not on intuition or speculation.

To a further extent, the results of this survey may be the subject of further research. In relation to basic statistics for healthcare in Greece, the data show there was a high concentration of experience, knowledge, and integrated care processes in regions with a large number of populations, e.g. Attica, Thessaloniki, and Larissa. There was a high experience of knowledge and management of patients with chronic diseases in the areas where there is a University Hospital and Medical School. There was a weakening of experience, knowledge and processes in regions with a high proportion of non-urban population and low purchasing power. In addition, public healthcare is weakening in regions with these characteristics. Regional (small) health structures (e.g. Health Centers, TOMY) find it particularly difficult to manage patients with chronic diseases. There was a high rate of care for children or dependent relatives (%), which affects families and encourages the implementation of integrated care. The number of public social care units governed by public law was particularly low in all the regions examined, regardless of their population.

## Conclusions

This study has provided valuable insights into the understanding, adoption, and barriers faced by healthcare professionals in implementing integrated care for chronic diseases in Greece. The findings shed light on key factors influencing the applicability of integrated care systems, which have significant implications for healthcare policymakers and stakeholders. Understanding the role of factors, such as gender, place of residence, professional employment, type of employment, professional status, higher education level, and attendance at relevant seminars, can help in developing targeted strategies and interventions to improve the implementation and adoption of integrated care systems. In addition, it provides valuable insights into the Greek health system considering available funding, accountability, delivery system design, community resources, and policies for tackling chronic diseases.

The varying levels of knowledge and experience with integrated care among healthcare professionals emphasize the need for targeted education and training initiatives to enhance understanding and foster the adoption of integrated care principles. The positive attitudes toward integrated care highlight its potential to improve patient outcomes, satisfaction, and overall healthcare quality in Greece.

However, the study also identified various barriers to implementing integrated care, including organizational, financial, regulatory, cultural, and individual factors. Addressing these barriers will require concerted efforts and collaboration among various stakeholders to create a supportive environment for integrated care implementation. Strategies may include allocating resources, promoting communication and collaboration between healthcare providers, and revising regulatory frameworks to facilitate integrated care practices.

Evidence has shown that the sustainability of healthcare systems can achieve better results if the treatment of chronic diseases is done in terms of prevention and health promotion. Care for people with chronic conditions should be shifted from acute care to a comprehensive, multidisciplinary, and cross-sectoral coordinated care program with the active involvement of patients.

In addition, the results show that micro-, meso- and macro-level problems can be addressed with an integrated care model for chronic diseases. The implementation of the model should start in the major cities of Greece (Athens and Thessaloniki) and possibly in other cities where there are university medical schools. This proposal is reinforced by the fact that university medical schools can contribute to the organization, education, and management of patients with chronic diseases, while there will also be benefits for research. The negative dynamics of chronic diseases in the Greek health system must be combined with a new public health approach focusing on the integrated development of humans in physical and mental health, according to the World Health Organization. The specific characteristics of Greek health personnel and the needs they need to meet require evidence-based health policies.

The research findings align with the Chronic Care Model, an evidence-based framework that emphasizes a proactive, patient-centered approach to chronic disease management. By addressing key areas of the model, such as self-management support, delivery system design, decision support, clinical information systems, organization of health care, and community resources, healthcare organizations can enhance chronic disease management and improve health outcomes for patients with chronic conditions.

While the study has some limitations, it contributes to the ongoing discussion on integrated care in Greece and provides direction for future policy and strategic initiatives. By identifying areas of focus and potential avenues for improvement, this study can help pave the way for a more coordinated, patient-centered, and high-quality healthcare system that effectively addresses the growing burden of chronic diseases in Greece. The research's implications extend beyond this study, and further research could explore the demand side of integrated care and address regional disparities in healthcare experiences and integrated care processes. By continuing to use evidence-based practices and promoting a culture of ongoing education and training for healthcare professionals, the healthcare system can continually improve chronic disease management and patient care in Greece.

## Appendices

**Table 6:** Multiple comparisons

Dependent variable: applicability of integrated care						
Bonferroni						
(I) Place of residence	(J) Place of residence	Mean Difference (I-J)	Std. Error	Sig.	95% Confidence Interval	
					Lower Bound	Upper Bound
Athens	Thessaloniki	0.04418	0.10260	1.000	-0.2032	0.2915
	Other city in Greece	0.21020	0.09001	0.061	-0.0068	0.4272
Thessaloniki	Athens	-0.04418	0.10260	1.000	-0.2915	0.2032
	Other city in Greece	0.16602	0.10343	0.329	-0.0833	0.4154
Other city in Greece	Athens	-0.21020	0.09001	0.061	-0.4272	0.0068
	Thessaloniki	-0.16602	0.10343	0.329	-0.4154	0.0833

**Table 7:** Multiple comparisons (Contd. 1)

Dependent variable: applicability of integrated care						
Bonferroni						
(I) Professional employment	(J) Professional employment	Mean Difference (I-J)	Std. Error	Sig.	95% Confidence Interval	
					Lower Bound	Upper Bound
Civil servant of a health service provider	Private employee of a health service provider	-0.21448	0.12736	0.280	-0.5215	0.0925
	Self-employed	-0.10345	0.09220	0.789	-0.3257	0.1188
Private employee of a health service provider	Civil servant of a health service provider	0.21448	0.12736	0.280	-0.0925	0.5215
	Self-employed	0.11102	0.14046	1.000	-0.2276	0.4496
Self-employed	Civil servant of a health service provider	0.10345	0.09220	0.789	-0.1188	0.3257
	Private employee of a health service provider	-0.11102	0.14046	1.000	-0.4496	0.2276

**Table 8:** Multiple comparisons (Contd. 2)

Dependent variable: applicability of integrated care						
Bonferroni						
(I) Type of work structure	(J) Type of work structure	Mean Difference (I-J)	Std. Error	Sig.	95% Confidence Interval	
					Lower Bound	Upper Bound
Public hospital	Private hospital	-0.16953	0.12795	1.000	-0.5099	0.1708
	Private doctor	-0.05851	0.09367	1.000	-0.3077	0.1907
	Health Center	0.31462	0.14161	0.163	-0.0621	0.6913
Private hospital	Public hospital	0.16953	0.12795	1.000	-0.1708	0.5099
	Private doctor	0.11102	0.13934	1.000	-0.2596	0.4817
	Health Center	0.48415^*^	0.17520	0.037	0.0181	0.9502
Private doctor	Public hospital	0.05851	0.09367	1.000	-0.1907	0.3077
	Private hospital	-0.11102	0.13934	1.000	-0.4817	0.2596
	Health Center	0.37312	0.15198	0.089	-0.0312	0.7774
Health Center	Public hospital	-0.31462	0.14161	0.163	-0.6913	0.0621
	Private hospital	-0.48415^*^	0.17520	0.037	-0.9502	-0.0181
	Private doctor	-0.37312	0.15198	0.089	-0.7774	0.0312

**Table 9:** Multiple comparisons (Contd. 3)

Dependent variable: applicability of integrated care						
Bonferroni						
(I) Professional status	(J) Professional status	Mean Difference (I-J)	Std. Error	Sig.	95% Confidence Interval	
					Lower Bound	Upper Bound
Doctor	Nurse	0.25195	0.08586	0.055	-0.0026	0.5065
	Psychologist	-0.03924	0.25474	1.000	-0.7945	0.7160
	Ergotherapist	-0.08826	0.35675	1.000	-1.1460	0.9695
	Social worker	-0.41179	0.25474	1.000	-1.1670	0.3435
	Other	-0.04904	0.27815	1.000	-0.8737	0.7756
Nurse	Doctor	-0.25195	0.08586	0.055	-0.5065	0.0026
	Psychologist	-0.29119	0.25930	1.000	-1.0600	0.4776
	Ergotherapist	-0.34021	0.36002	1.000	-1.4076	0.7272
	Social worker	-0.66374	0.25930	0.166	-1.4325	0.1051
	Other	-0.30099	0.28234	1.000	-1.1381	0.5361
Psychologist	Doctor	0.03924	0.25474	1.000	-0.7160	0.7945
	Nurse	0.29119	0.25930	1.000	-0.4776	1.0600
	Ergotherapist	-0.04902	0.43259	1.000	-1.3316	1.2336
	Social worker	-0.37255	0.35321	1.000	-1.4198	0.6747
	Other	-0.00980	0.37045	1.000	-1.1081	1.0885

**Table 10:** Multiple comparisons (Contd. 4)

Dependent variable: applicability of integrated care						
Bonferroni						
(I) Higher education level	(J) Higher education level	Mean Difference (I-J)	Std. Error	Sig.	95% Confidence Interval	
					Lower Bound	Upper Bound
Basic degree	Master	-0.18073	0.09293	0.318	-0.4279	0.0665
	Phd	-0.35057^*^	0.10385	0.005	-0.6268	-0.0743
	Postdoc	-0.25240	0.19646	1.000	-0.7750	0.2702
Master	Basic degree	0.18073	0.09293	0.318	-0.0665	0.4279
	Phd	-0.16984	0.10045	0.553	-0.4370	0.0974
	Postdoc	-0.07167	0.19468	1.000	-0.5896	0.4462
Phd	Basic degree	0.35057^*^	0.10385	0.005	0.0743	0.6268
	Master	0.16984	0.10045	0.553	-0.0974	0.4370
	Postdoc	0.09818	0.20013	1.000	-0.4342	0.6305
Postdoc	Basic degree	0.25240	0.19646	1.000	-0.2702	0.7750
	Master	0.07167	0.19468	1.000	-0.4462	0.5896
	Phd	-0.09818	0.20013	1.000	-0.6305	0.4342
